# NUrsing Homes End of Life care Program (NUHELP): developing a complex intervention

**DOI:** 10.1186/s12904-021-00788-1

**Published:** 2021-06-26

**Authors:** Emilio Mota-Romero, Ana Alejandra Esteban-Burgos, Daniel Puente-Fernández, María Paz García-Caro, Cesar Hueso-Montoro, Raquel Mercedes Herrero-Hahn, Rafael Montoya-Juárez

**Affiliations:** 1 Salvador Caballero Primary Care Centre, Andalusian Health Service, Granada, Spain; 2 Department of Nursing, Doctoral Program in Clinical Medicine and Public Health, University of Granada, Granada, Spain; 3 Doctoral Program in Clinical Medicine and Public Health, University of Granada, Granada, Spain; 4 Department of Nursing, Brain and Behaviour Research Institute, University of Granada, Mind, Spain; 5 Department of Nursing, University of Granada, Granada, Spain; 6 University Health Provider Institution, University of Antioquia, Medellín, Colombia

**Keywords:** Nursing Home Care, End of Life, Holistic Care, Nurse-Patient interaction, Nurse-Patient Relationships, Older People, Palliative Care

## Abstract

**Background:**

Nursing homes are likely to become increasingly important as end-of-life care facilities. Previous studies indicate that individuals residing in these facilities have a high prevalence of end-of-life symptoms and a significant need for palliative care. The aim of this study was to develop an end-of-life care program for nursing homes in Spain based on previous models yet adapted to the specific context and the needs of staff in nursing homes in the country.

**Methods:**

A descriptive study of a complex intervention procedure was developed. The study consisted of three phases. The first phase was a prospective study assessing self-efficacy in palliative care (using the SEPC scale) and attitudes towards end-of-life care (using the FATCOD-B scale) among nursing home staff before and after the completion of a basic palliative care training program. In the second phase, objectives were selected using the Delphi consensus technique, where nursing home and primary care professionals assessed the relevance, feasibility, and level of attainment of 42 quality standards. In phase 3, interventions were selected for these objectives through two focus group sessions involving nursing home, primary care, and palliative care professionals.

**Results:**

As a result of the training, an improvement in self-efficacy and attitudes towards end-of-life care was observed. In phase 2, 14 standards were selected and grouped into 5 objectives: to conduct a comprehensive assessment and develop a personalized care plan adapted to the palliative needs detected; to provide information in a clear and accessible way; to request and record advance care directives; to provide early care with respect to loss and grief; to refer patients to a specialized palliative care unit if appropriate, depending on the complexity of the palliative care required. Based on these objectives, the participants in the focus group sessions designed the 22 interventions that make up the program.

**Conclusions:**

The objectives and interventions of the NUHELP program constitute an end-of-life care program which can be implemented in nursing homes to improve the quality of end-of-life care in these facilities by modifying their clinical practice, organization, and relationship with the health system as well as serving as an example of an effective health intervention program.

**Supplementary Information:**

The online version contains supplementary material available at 10.1186/s12904-021-00788-1.

## Background

The aging of the population has led to an increase in chronic disease [1]. To address this situation, institutions such as the World Health Organization recommend the use of strategies such as palliative care, particularly among people over 65 years of age [1]. Palliative care is interdisciplinary care that focuses on improving the quality of life of individuals of any age living with a life-threatening illness, as well as that of their families [2]. This approach to palliative care transcends mere symptom control, although the latter is also of paramount importance [1].

Despite being one of the groups that would benefit most from this approach, elderly people receive very little palliative care, since this type of care has traditionally been aimed at cancer patients and healthcare professionals receive insufficient training in managing comorbidities in this age group [3]. Another factor that may lead to reduced access to palliative care for the elderly is the lack of integration between nursing homes and health systems in different countries [4].

Indeed, the white paper by the European Association of Palliative Care [5] and the results reported by the European PACE project (PAlliative Care for older people in care and nursing homes in Europe) [6] emphasize the importance of intervention programs that take into account specific cultural and organizational contexts and involve participation by the professionals working at the institutions where the interventions are to be implemented.

Nursing homes are likely to become increasingly important as end-of-life care facilities. Previous studies indicate that individuals residing in these facilities have a high prevalence of end-of-life symptoms and a significant need for palliative care [7–12].

Various projects and interventions have been developed to improve the provision of end-of-life care in nursing homes such as the Gold Standards Framework for care homes [13, 14], the Route to Success program [15], or the Namaste program [16], which is specifically designed for patients with dementia. Recently, the European PACE project tested the effectiveness of an end-of-life intervention program in nursing homes in seven countries, among which Spain was not included [17].

These intervention programs share several common characteristics. A recent literature review [18] stresses that, in order to implement end-of-life care interventions in nursing homes, a capacity building approach is needed in addition to training in palliative care. This approach includes an internal team with effective leadership, support from an external palliative care team, and constant communication between the two teams regarding specific cases where necessary.

After reviewing the literature on the experiences of nursing home staff, Bolt et al. (2019) [19] reported that healthcare institutions should facilitate the implementation of interventions in these facilities, while focusing on the needs of staff at all times. Specifically, the authors pointed out that improvement is needed in end-of-life care programs in areas such as spirituality, advance directive planning, and family involvement.

Members of the PACE project have published a review echoing the most effective strategies for implementing end-of-life care programs in nursing homes, highlighting the fundamental role of the particular context, the involvement of the center’s own professionals, and its culture in relation to palliative care [6]. These same points were noted in the white paper from the European Association of Palliative Care [5].

In Spain, as in other European countries, a high prevalence of palliative care needs and difficult-to-control symptoms have also been reported in nursing homes [20–22]. However, no palliative care programs specifically focused on this type of facility have been published to date [23].

The emergence of the current COVID-19 pandemic has brought to light the shortcomings of the care provided in nursing homes. Despite the dramatic numbers of elderly people who have died or been infected in several countries bordering Spain, this crisis may represent an opportunity to develop a better model of care in these institutions [24]. This model of care must strike a balance between disease prevention and a more humane approach that addresses the psychosocial needs of the elderly [25]. Palliative care is an essential part of this approach. However, a recent review [26] has noted that, although a wide range of recommendations for preventing and treating patients with COVID-19 in nursing homes have been published, few homes have taken the time to address the main recommendations regarding palliative care needs.

The aim of this study was to develop an end-of-life care program for nursing homes in Spain based on previous models yet adapted to the specific context and the needs of staff in nursing homes in the country. The specific objectives for each phase were:

- Phase 1: To increase self-efficacy in palliative care and improve attitudes toward end-of-life care among professionals working in the participating nursing homes.
- Phase 2: To select objectives that are relevant, feasible, and capable of generating changes in end-of-life care in nursing homes.
- Phase 3: To select the most suitable intervention to achieve each objective based on the experience of each institution.

## Methods

### Context and target population

The program was developed for 8 Andalusian nursing homes which were selected based on their institutional characteristics. Nursing homes in Spain offer all-inclusive accommodation to dependent individuals on a temporary or permanent basis. The objectives of this type of facility are to improve residents’ quality of life and promote their individual autonomy through the provision of intervention programs and activities tailored to their specific needs [27]. They all had more than 60 beds and were privately funded. In Spain, there were a total of 3844 private nursing homes (71.53 %) in 2017. Private institutions usually offer a number of beds in conjunction with the public health system [28]. Complex care services are offered depending on the number of beds. Nursing homes with more than 60 beds are required to offer 24-hour nursing services and their own medical care [29]. The public, universal nature of healthcare in Spain means that every nursing home is supervised by a primary care team (a physician and a nurse) depending on the geographic area in which the nursing home is located. Each nursing home has its own contracted professionals (a physical therapist, a psychologist, an occupational therapist, a social worker, a nursing assistant, and nurses) and a primary care team assigned by the public health system.

To select the nursing homes included in this study, a list of facilities meeting the inclusion criteria (i.e. presence of a multidisciplinary team, professionals interested in participating in the study, and the availability of public and private beds) was requested from the primary care district in the public health system. Each facility was then contacted and an interview was arranged to inform them about the project. Finally, the nursing homes that agreed to carry out the intervention program were included in the study.

### Intervention development approach

The care program to be developed as part of this study may be described as a complex intervention as it is made up of various interacting components. Complexity may be caused by the presence of several potential outcomes, variability in the target population, or a number of elements in the intervention package itself [30, 31]. The intervention program to be developed must focus on multiple populations (professionals, family members, residents) and may have a large number of potential components and outcome variables.

This study addresses many aspects of the development phase of a complex intervention, including the exploration of relevant frameworks, identification of existing evidence, exploration of potential intervention components, and modeling of the intervention components [30, 31]. The implementation process has already begun and will be described in subsequent studies. The development process combines both qualitative and quantitative methods, as proposed in similar settings [31, 32].

In addition, recommendations from specific literature reviews on interventions in nursing homes have been followed, as explained in the introduction [6, 18, 19].

The development of the program can be divided into three phases. The design, participants, and methodology of each of the three phases are shown below. A summary of the methodology, the participants, and the main outcomes of each phase is shown in Table 1. The study was approved by the Research Ethics Committee “Comités de Ética Asistencial Granada Metropolitano” for the Andalusian Public Health System with reference number (0706-N-17).

**Table 1:** Summary of the objectives and methodology of the different phases in the NUHELP program

Phase	Phase 1: Training	Phase 2: Selection of the objectives	Phase 3: Selection of the interventions
**Objectives**	To increase self-efficacy in palliative care and improve attitudes towards end-of-life care among professionals working at the participating nursing homes.	To select objectives that are relevant, feasible, and capable of generating changes to end-of-life care in nursing homes.	To select the most suitable intervention interventions to achieve the objectives in phase 2 based on the experience of each institution.
**Participants**	54 nursing home professionals	52 professionals (38 from nursing homes and 14 from primary care)	25 professionals (8 from nursing homes, 8 from primary care, 4 nursing home coordinators, and 5 researchers)
**Methodology**	Prospective study	Delphi panel of experts	2 sessions held with 5 focus groups (5 participants). The focus group topics were as follows: assessment, information, advance care directives, grief and emotion management, and referral.
**Schedule**	6 months (September 2017- January 2018)	4 months (February-May 2018)	6 months (October 2018-May 2019)

### Phase 1: Palliative care training in participating nursing homes

#### Design of the phase 1

Following the recommendations made by Hockley et al. [33], basic training in palliative care was provided to nursing home staff. The design of this phase was a prospective study assessing the efficacy of the training delivered. The effectiveness of the training program was assessed pre/post intervention.

#### Participants

The training was intended for professionals from the nursing homes participating in the project (physicians, nurses, social workers, psychologists, physical therapists, and occupational therapists).

#### Data collection

The 50-hour online training course was taught by professionals from the University of Granada, Spain, and the Andalusian Public Health System between September 2017 and January 2018. The online course contained 7 modules featuring scientific literature, presentations, videos, case studies, and a discussion forum. Before passing a module and moving on to the next, participants had to pass an exam and complete a number of activities. The course was open for completion for 5 months. The course was accredited by the Continuous Training Commission of the Spanish National Health System and the Andalusian Agency for Healthcare Quality. The training included:

1. General aspects of palliative care, models of care, and identification of palliative care needs.
2. Principles of symptom control and comfort care. Comprehensive assessment and pain control.
3. Monitoring of nutrition, excretion, activity/rest, and cognitive symptoms.
4. Psychosocial care in palliative care.
5. Peri-death care, emergency care, and special end-of-life situations.
6. Communication and decision-making.
7. Grief care and burnout prevention in healthcare professionals.

#### Instruments

Changes in perceptions of efficacy among the professionals themselves were assessed using the Self-Efficacy in Palliative Care (SEPC) scale [34]. This scale is based on the theoretical principles of Bandura’s Social Cognitive Theory [35] and consists of 23 items that assess perceived efficacy in relation to communication (8 items), patient management (8 items), and multiprofessional teamworking (7 items). Each behavior or skill was assessed using a 100 mm visual analogue scale ranging from “very anxious” to “very confident.” The reliability and validity of the Spanish version of the scale were determined for nursing professionals and students, yielding a Cronbach’s *α* value greater than 0.944 on all subscales [36].

Changes in attitudes towards end-of-life care were assessed using the FATCOD scale, designed by Frommelt in 1991 [37] to evaluate nurses’ attitudes towards the care of terminally ill patients and their families. A new version (FATCOD-B) was subsequently developed by the same author in 2003, allowing the scale to be used among different healthcare professionals [38]. The FATCOD-B scale showed an inter-rater agreement of 1.00 and a Pearson’s test-retest of 0.9269. The scale consists of 30 items rated on a 5-point Likert scale, 15 of which are reversely worded.

#### Data analysis

The SEPC and FATCOD-B scales were administered at the beginning and end of the training course. To assess the changes produced by the intervention, non-parametric tests were performed (Wilcoxon’s test for related samples) and statistical results were shown in terms of median and interquartile range. The magnitude of the effect was also calculated for each of the dimensions related to the training course.

Phase 2: Selection of the objectives.

#### Design

The objectives were selected using an adaptation of the Delphi consensus technique.

#### Participants

Five professionals from each nursing home who had completed the training course in the previous phase, as well as professionals (a physician and a nurse) from the referral primary care centers for each nursing home were selected.

#### Data collection

Each standard was assessed based on three dimensions: its relevance to the nursing home setting; the feasibility of its implementation; and its level of attainment in each nursing home. Each dimension was rated on a Likert scale with options ranging from 1 (not at all) to 5 (absolutely). Two consecutive Delphi rounds were held. Between the two rounds, each participant was provided with a summary of the responses of all participants, in addition to their own previous responses.

#### Instruments

As a starting point, 42 standards that could be applicable to nursing homes were selected from *End of Life Care for Adults* by the British National Institute for Health and Care Excellence, NICE [39], and the New Health Foundation [40]. A native Spanish translator translated them into Spanish and another translator translated them back into English in order to ensure that the original meaning was preserved.

#### Data analysis

The objectives were selected using a cascading model by applying the criteria in a consecutive manner, as shown in Fig. 1. For the dimensions of relevance and feasibility, the standards selected were those rated as 4 or 5 by 70 % of the participants. This level of agreement was approved by the research team prior to the start of the Delphi group [41]. Given that one of the specific objectives of this study was to develop an intervention program that would generate changes to end-of-life care in nursing homes, the standards selected were those that did not exceed 70 % of the sum of 4 and 5 s with regard to the level of attainment in each nursing home. Due to the lack of formal records on most of the aspects to be evaluated, the evaluation was carried out using subjective, individual assessments from the professionals. Finally, some of the standards selected were merged with one another in view of their similarity and were worded in such a way as to make them attainable.

### Phase 3: Selection of the interventions

#### Design

Consecutive focus group sessions with professionals from participating nursing homes.

#### Participants

 The following individuals participated in this phase: one representative of the professionals from each nursing home, one representative of the referral primary care centers for the nursing homes, the coordinators of the nursing homes in the districts of Granada and Jaén, and physicians specializing in palliative care. In total, 25 professionals participated (8 from nursing homes, 8 from primary care centers, 4 nursing home coordinators, and 5 researchers).

#### Data collection

Based on the objectives selected in phase two, focus groups of five participants were established. In the focus group sessions for objectives 1–4 (assessment, information, grief, and emotion management), 2 nursing home professionals and 2 primary care professionals participated. Given the complexity of objective 5 (referral), it was assigned to the nursing home coordinators and palliative care physicians. One researcher would act as the moderator and record the relevant information in each focus group session. Two sessions were held for each focus group, with a duration of approximately one hour.

During the first session, in October 2018, the professionals discussed the interventions that were being implemented in their centers, the outcomes they had attained, the difficulties they had encountered, and how they had overcome them.

To facilitate consensus-building on the interventions to be implemented, participants were provided with two documents one week prior to the first session: (1) one international literature review of interventions implemented in nursing homes for each of the objectives proposed and (2) a report on the interventions carried out in the participating centers for each of the objectives, which had been collected previously via an online form.

After the transcripts had been analyzed, the proposals for each objective were synthesized and sent back to the members of each group.

In the second session, held in May 2019, the proposals agreed upon in session 1 were discussed in order to adapt them to the clinical reality of the nursing homes in the study. The script of the two sessions is shown in Table 2.

**Table 2:** Script for focus group sessions

Session 1	Session 2
What are you doing at your nursing home with respect to this objective?	Which of the interventions mentioned in the documentation provided do you think is most appropriate for implementation at the centers? If none of them seems suitable, what would you propose?
How would you assess it?	What preconditions would need to be in place for the intervention to be properly implemented?
What results have you achieved in this regard?	Which professionals would be best suited to carry out the intervention at the centers?
What difficulties have you encountered with this procedure?	Where (in which space) should the intervention take place?
What would you improve about this procedure and how?	What time (or times) are best for implementing the intervention?
Please indicate any other aspects you would like to add or elaborate on.	What other aspects should be taken into account for the intervention to be successful?
	How could the intervention be assessed for effectiveness? In other words, how can one tell if the objective has been achieved?

 After the focus group sessions were held, the researchers synthesized the proposals made and forwarded them to the participants in each group for final approval. This article focuses on the interventions that were finally selected.

### Ethical considerations

This study complies with the basic ethical principles governing responsible conduct in research involving human subjects. Informed consent was sought from all participants. The study was approved by the Research Ethics Committee (0706-N-17). The patients’ data were anonymized in compliance with Spanish regulations.

## Results/findings

### Phase 1: Training

Fifty-two professionals from the nursing homes received the training, of which 27 were nurses (51.9 %), 7 were occupational therapists (13.6 %), 6 were psychologists (11.5 %), 5 were social workers (9.6 %), 5 were physical therapists (9.6 %), and 2 were physicians (3.8 %). An increase in all variables was observed following the training, both for the FATCOD-B and SEPC scales and for the different subscales of the SEPC scale (teamwork, communication, and psychosocial/spiritual and physical aspects of patient management). Effect sizes of 0.710 and 1.5 were observed for the total scores on the SEPC and FATCOD-B scales, respectively. The results of the training course is shown in Table 3.

**Table 3:** Results of the training course

	Pre-test			Pre-test			*p*	*r*
Tools	Me	R	IQR	Me	R	IQR		
SEPC	6.13	5.22	2.05	8.00	5.78	**1.76**	**0.000**	**0.710**
Factor 1: multidisciplinary teamwork	6.57	6.14	2.1	8.14	5.14	**1.85**	**0.000**	**1.420**
Factor 2: communication	6.31	6.50	2.34	8.00	7.00	**1.71**	**0.000**	**0.706**
Factor 3: patient management - physical	6.20	7.20	2.30	8.00	7.2	**2.1**	**0.000**	**1.410**
Factor 4: patient management - psychosocial/spiritual	6.16	7.00	2.00	7.66	2.58	**1.86**	**0.000**	**0.727**
FATCOD-B	127.00	47.00	11	133.50	56.90	**16.53**	**0.003**	**1.5**

Wilcoxon’s test for related samples

*r *magnitude of the effect

Me  median

R range

IQR interquartile range

### Phase 2: Selection of the objectives

For the selection of the objectives, 40 professionals from the participating nursing homes who had completed the training course in the previous phase were contacted (5 professionals per center). Referral primary care physicians and nurses for each nursing home were also contacted (16 in total). In total, 52 participants responded, with 38 and 14 participants from each group respectively (response rate = 93 %).

On the basis of the established criteria, the professionals from the nursing homes considered the 42 initial standards to be relevant, but only 28 of them were considered to be feasible. Of these, 14 were considered to have a low level of attainment in the participating centers. The scores given by nursing home professionals to the standards for each dimension in the last Delphi round are shown as Supplementary Material 1. Finally, the 14 standards were reworded and merged into five objectives in view of their similarity. The five objectives were as follows: “To conduct a comprehensive assessment and develop a personalized care plan adapted to the palliative needs detected”, “To provide information in a clear and accessible way”, “To request and record advance care directives”, “To provide early care with respect to loss and grief”, and “To refer patients to a specialized palliative care unit if appropriate, depending on the complexity of the palliative care required”.

### Phase 3: Selection of the interventions

The interventions proposed were discussed in the focus group sessions. Following these discussions, the participants selected 22 interventions, which are shown in Table 4. Some interventions were described as optional by the participants, since their implementation depended on residents displaying certain characteristics. An example of an intervention is shown in supplementary material 2.

**Table 4:** Relationships between the standards, objectives, and interventions in the NUHELP program

Objectives and standards:	Interventions
**Objective 1: To conduct a comprehensive assessment and develop a personalized care plan adapted to the palliative needs detected.** Standards on which this objective is based: • People approaching the end of life are offered comprehensive assessments in response to their changing needs and preferences. • A personalized care plan for people approaching the end of life which is appropriate to their needs and preferences is developed and reviewed.	-Palliative care needs are identified.
	-A comprehensive geriatric and palliative care assessment is conducted.
	-A personalized care plan is created and adapted to the palliative care needs identified.
**Objective 2: To provide information in a clear and accessible way.** Standards on which this objective is based: • The professionals on the team ask the patient and family members how they would like to be informed about the diagnosis/prognosis/treatment progress of the disease and reflect this in the clinical record in a clearly visible place. • People approaching the end of life receive communication and information in an accessible and sensitive way in response to their needs and preferences. • The team provides information on the benefits and adverse effects of the treatments that may be provided to the patient. • The team enables the patient to be involved in decision-making throughout the course of the disease.	-The information that the patient and/or family have regarding the patient’s clinical status is ascertained.
	-The patient’s preferences regarding the information they wish to receive are explored.
	-The family’s preferences regarding the information provided to them and to the patient are explored when there is no secrecy surrounding the patient’s health.*
	-The family’s preferences regarding the information provided to them and to the patient are explored when there is secrecy surrounding the patient’s health.*
	-The patient and/or family are informed about clinical matters.
**Objective 3: To request and record advance care directives.** Standards on which this objective is based: • There is an advance care directive document in place. • The team enables the patient to be involved in decision-making throughout the course of the disease.	-The existence of documents stating the patient’s preferences is verified.
	-The patient’s preferences regarding decision-making are assessed (patients without cognitive impairment).*
	-Information is provided on what advance care directives are and what their purpose is.
	-The advance care directive document is discussed with the patient and/or family. +
	-The advance care directive document is filled out. +
	-The decisions made are reported to primary care workers and to the members of the healthcare team at the nursing home. +
**Objective 4**: T**o provide early care with respect to loss and grief.** Standards on which this objective is based: • Families of the deceased are offered emotional and spiritual support appropriate to their needs and preferences during the grieving process. • Families of the deceased are offered emotional and spiritual support appropriate to their needs and preferences during the grieving process.	-Family involvement in the patient’s care is encouraged.
	-Communication between the resident and the family is encouraged.
	-Risk factors for complicated grief are identified and addressed.
	-The patient’s spiritual needs are valued.*
**Objective 5: To refer patients to a specialized palliative care unit if appropriate, depending on the complexity of the palliative care required.** Standards on which this objective is based: • The clinical material and medication needed to carry out care work are available to staff. • Patient referral criteria are clearly defined. • People approaching the end of life who may benefit from specialist palliative care are offered this care in a timely manner appropriate to their needs and preferences, at any time of day or night. • People approaching the end of life who experience a crisis at any time of day or night receive prompt, safe, and effective urgent care appropriate to their needs and preferences.	-The nursing home has established priority levels for the provision of specialist palliative care resources.
	-Priority for providing these resources is established based on residents’ palliative care needs and on complex and highly complex palliative care aspects.
	-There is a procedure in place to request the provision of specialist palliative care resources.
	-The interventions and care recommended by the support team are provided and the situation is re-assessed whenever necessary or recommended.
*Depending on the presence or absence of cognitive impairment. +Depending on whether they wish to proceed.	

## Discussion

This paper shows the development of a complex intervention: an end-of-life care program for patients in Spanish nursing homes. The NUHELP program has been shaped and adapted to this context in accordance with previous models, based on advice from primary care professionals responsible for the care provided at the participating nursing homes and designed to meet the needs of professionals at the homes. It is worth noting that this is the first end-of-life intervention program developed in nursing homes in Spain. Eleven out of the 42 preexisting palliative care standards were selected and grouped into 5 objectives, for which 22 interventions were created.

 Firstly, a core element in the development of the program was training at nursing homes, which was intended to increase self-efficacy in palliative care and improve the attitudes of nursing home professionals towards end-of-life care. As noted by Honinx et al. [42], for interventions to be successful, it is necessary to improve the training of nursing home professionals in palliative care. The project PACE [43] concluded that greater efforts were needed to increase understanding of palliative care in these institutions, albeit with different training strategies required depending on the country.

 In line with our initial hypothesis, following the palliative care training course delivered to multidisciplinary teams at the nursing homes, an improvement in their efficacy in terms of communication, management, and multidisciplinary teamwork was observed, as well as an improvement in their attitudes towards end-of-life care. Our results corroborate those of other studies, which conclude that training in nursing homes improves not only end-of-life competencies, but also attitudes towards end-of-life care, showing an effect size with a large magnitude for both competencies and attitudes [44, 45].

In addition, the selection process using the Delphi technique yielded 11 of the 42 preexisting standards that were reworded and merged together into 5 program objectives. In order to select them, the relevance of the standards, their feasibility, and their level of attainment based on the resources of each nursing home were taken into account. Being aware of the limitations of a particular setting is key to ensuring the success of a complex intervention such as this, as recommended in recently published reviews [19].

Among all the standards that the professionals rated as feasible, the standards that had a low level of attainment at the centers were chosen. Some of the objectives selected had already been identified in previous studies, while others reflect the unique characteristics of nursing homes in Spain.

Regarding the first objective selected, “to conduct a comprehensive assessment and develop a personalized care plan adapted to the palliative needs detected”, a proper assessment of palliative needs and individualized care planning is the basis for successful interventions. Previous studies show that comprehensive assessment of older adults improves their functional status and reduces the number of hospitalizations and length of hospital stays [46]. This is because potential complications that may be developing in frail, older individuals are detected earlier [47]. Similar results were observed in Spanish nursing homes where comprehensive geriatric assessment, among other factors, made it possible to reduce hospital and emergency room visits in institutionalized patients and to optimize pharmacy costs [48].

Of the five objectives of the NUHELP program, both the second objective (“to provide information in a clear and accessible way”) and the third objective (“to request and record advance care directives”) are linked to information and decision-making. Highlighting end-of-life information as an area for improvement in nursing homes [49] is expected to facilitate professionals’ end-of-life conversations with residents and family members in a proactive manner, as well as address unavoidable decisions in accordance with residents’ wishes and needs [50].

 As a result, the third objective of the NUHELP program focused on developing specific programs to help implement advance care directives in nursing homes. These programs focus not only on discussing specific clinical or treatment issues, but also on discussing patients’ values, beliefs, and goals with the patients themselves and their families in order to assist with clinical decision-making in the event that the patient is unable to make these decisions on their own [51–54].

The fourth objective of the NUHELP program proposes “to provide early care with respect to loss and grief”. Several factors highlight the importance of dealing with anticipatory grief in nursing homes: ambivalence between caregivers’ desire to take a break from caring for the patient, the desire to avoid their death, and emotional dependence on the patient can lead to subsequent grief complications [55]. Acceptance of death by both parties can help prioritize decision-making, prioritize patient comfort over longevity, and help family members deal with the subsequent loss [56, 57]. Improved family communication can also make it easier to say goodbye to loved ones [55]. This is why the NUHELP program includes interventions to improve family members’ involvement in the daily care of institutionalized patients.

Spiritual and religious support for individuals approaching the end of their lives and their families was also considered a priority intervention for this objective. Proper management of this dimension may improve wellbeing and relieve pain and other symptoms [58], highlighting the need for correct identification of needs in this area. It has been noted that improved access to religious services offered by many nursing homes can help to meet the spiritual needs of their residents [59]. As for specific interventions to improve spirituality, there are a small number of clinical trials on interventions in spirituality, and the studies published are extremely heterogeneous [60].

The fifth and final objective of the NUHELP program is “to refer patients to a specialized palliative care unit if appropriate, depending on the complexity of the palliative care required”. Referring patients from nursing homes to specialized palliative care services, even if the resident remains in the nursing home (specialist palliative care consultations), is expected to reduce the number of hospitalizations and visits by emergency care teams [61]. According to a recent review [62], timely identification of the patients who would benefit most from these services in nursing home end-of-life care programs would improve referral when needed and encourage appropriate use of available resources.

Moreover, given the heterogeneity of the resources for end-of-life care available at each nursing home, each institution needs to assess the circumstances in which it would refer patients requiring complex care. The NUHELP program includes this intervention within objective 5.

The results of this study provide an example of the creation of a complex, comprehensive intervention program for Spanish nursing homes by highlighting their conditions, strengths, and needs and addressing them through a set of interventions aimed at providing high-quality basic care and enhancing coordination with the public health system.

Among the limitations of the present study, it should be noted that the selection of the centers and participants throughout the different phases could not be randomized. As a result, it is likely that the nursing homes and professionals involved were the most motivated to participate. Furthermore, although the literature indicates that all nursing homes in Spain could benefit from this program, it has been specifically designed by the participating nursing homes and adapted to their particular characteristics, so caution should be exercised when extrapolating results.

## Conclusions

The NUHELP program has been developed using a combination of quantitative and qualitative methods. 22 interventions were selected to enable the attainment of the 5 objectives in the intervention program and improve end-of-life care at these centers.

This intervention program aimed to improve the basic palliative care provided at the different nursing homes by modifying their clinical and organizational practice as well as their relationship with the public health system, presenting palliative care as a necessity at these centers and providing tools for successful palliative care delivery. The NUHELP program could also be used as an example of complex intervention development when designing other programs at nursing homes or other types of facilities.

## Supplementary Information

**Additional file 1.** Scores given by nursing home professionals to the standards for each dimension in the second Delphi round.

**Additional file 2. **Example of NUHELP Intervention.

## Data Availability

The datasets during and/or analyzed during the current study available from the corresponding author on reasonable request.

## Abbreviations

SEPC: Self-Efficacy in Palliative Care
FATCOD-B: Frommelt Attitudes Toward Care of the Dying Scale Form B
NUHELP: NUrsing Homes End-of-Life care Program
PACE: PAlliative Care for older people in care and nursing homes in Europe
COVID-19: coronavirus disease 2019
NICE: British National Institute for Health and Care Excellence.
IQR: interquartile range
Me: median
R: range
