# Supplementary material for: NUrsing Homes End of Life care Program (NUHELP): developing a complex intervention
Source: BMC Palliat Care. 2021 Jun 26;20:98. doi: 10.1186/s12904-021-00788-1 (PMC8234765; doi:10.1186/s12904-021-00788-1)
Supplement: Supplementary file 1 — Additional file 1. Scores given by nursing home professionals to the standards for each dimension in the second Delphi round. [file 12904_2021_788_MOESM1_ESM.docx]

Supplementary material: Scores given by nursing home professionals to the standards for each dimension in the second Delphi round.

|  |  | RELEVANCE | | FEASIBILITY | | LEVEL OF ATTAINMENT | |
| --- | --- | --- | --- | --- | --- | --- | --- |
| Source | QUALITY STANDARDS | *M* (SD) | %  (4-5) | *M* (SD) | %  (4-5) | *M* (SD) | %  (4-5) |
| NICE 15 | Health and social care workers have the knowledge, skills, and attitudes necessary to be competent to provide high-quality care and support for people approaching the end of life and their families and carers. | 4.471  (0.7308) | **90.2*** | 3.961  (1.019) | **74.5*** | 3.838  (0.8665) | **69.5*** |
| NICE 16 | Generalist and specialist services providing care for people approaching the end of life and their families and carers have a multidisciplinary workforce sufficient in number and skill mix to provide high-quality care and support. | 4.353  (0.8444) | 80.4 | 3.686  (0.9693) | 51 | 3.514  (0.9609) | 48.6 |
| NHF 3.5 | The roles and competencies of all professionals on the palliative care team are clearly defined. | 4.412  (1.1209) | **90.2*** | 4.059  (1.1209) | **78.4*** | 3.703  (1.0237) | **62.1*** |
| NHF 5.7 | The interdisciplinary team coordinates and works together on case conferences for each of the cases treated by the team. | 4.471  (0.7308) | 86.3 | 3.725  (1.0213) | 60.8 | 3.486  (1.07) | 51.3 |
| NHF 12.6 | The criteria for referral to other professionals in the team/center are clearly defined: criteria for care by the psychologist, criteria for care by the social worker, criteria for care by the counselor or spiritual guide. | 4.216  (0.9233) | 80.4 | 3.882  (1.0516) | 68.3 | 3.649  (1.0857) | 62.1 |
| NH 2.3 | The clinical material and medication needed to carry out care work are available to staff. | 4.412  (0.8289) | **86.3*** | 3.941  (0.8345) | **74.5*** | 4.36 (0.957) | **68.4*** |
| NHF 2.1 | The meeting rooms, offices, etc. necessary for carrying out care activities are available to staff. | 4.510  (0.6441) | **92.1*** | 4.373  (0.8709) | **88.2*** | 4.03  (1.1184) | 78.4 |
| NICE 1 | People approaching the end of life are identified in a timely manner. | 4.608  (0.6026) | **94.2*** | 4.353  (0.7162) | **86.3*** | 4.297  (0.7018) | 86.4 |
| NICE 10 | People approaching the end of life who may benefit from specialist palliative care are offered this care in a timely manner appropriate to their needs and preferences, at any time of day or night. | 4.510  (0.6745) | **94.1*** | 3.902  (0.9435) | **72.5*** | 3.784  (0.9170) | 68.5* |
| NICE 3 | People approaching the end of life are offered comprehensive assessments in response to their changing needs and preferences. | 4.490  (0.7314) | **90.2*** | 4.157  (0.8573) | **78.5*** | 3.865  (0.9178) | **67.5*** |
| NICE 7 | Families and carers of people approaching the end of life are offered comprehensive assessments in response to their changing needs and preferences. | 4.275  (0.9398) | 84.3 | 3.686  (1.0294) | 62.8 | 3.514  (1.0960) | 56.7 |
| NHF 12.4.12 | The assessments made by the interdisciplinary team are continuously monitored. | 4.431  (0.7812) | 86.3 | 3.941  (0.9677) | 62.8 | 3.811  (1.1015) | 62.1 |
| NICE 4 | People approaching the end of life have their physical needs safely, effectively, and appropriately met at any time of day or night, including access to medicines and equipment. | 4.569  (0.6710) | **89.5*** | 4.039  (0.8237) | **76.5*** | 4.027  (0.6866) | 83.8 |
| NICE 4 | People approaching the end of life have their psychological needs safely, effectively, and appropriately met at any time of day or night, including access to medicines and equipment. | 4.294  (0.9010) | 92.3 | 3.412  (1.0616) | 47 | 3.270  (1.0710) | 48.6 |
| NICE 5 | People approaching the end of life are offered social and practical support which is appropriate to their preferences and maximizes independence and social participation for as long as possible. | 4.275  (0.9608) | 82.3 | 3.765  (1.1241) | 62.8 | 3.514  (1.1931) | 59.4 |
| NICE 6 | People approaching the end of life are offered spiritual and religious support appropriate to their needs and preferences. | 4.255 (1.0167) | 78.4* | 4.012 (1.1544) | 70.2* | 3.703  (1.2217) | 67.5 |
| NICE 7 | Families and carers of people approaching the end of life are offered comprehensive support in response to their changing needs and preferences. | 4.353  (0.8905) | 84.4 | 3.804  (0.9802) | 66.7 | 3.676  (0.9734) | 62.1 |
| NHF13.1 | The team engages with family members in patients’ care. | 4.549  (0.6727) | **94.1*** | 4.118  (0.8160) | **80.4*** | 4.00  (0.7454) | 78.4 |
| NICE 8 | People approaching the end of life receive consistent care that is coordinated effectively across all relevant settings and services at any time of day or night. | 4.373  (0.8237) | 86.3 | 3.784  (1.0063) | 50.9 | 3.730  (0.9617) | 67.5 |
| NICE 9 | People approaching the end of life who experience a crisis at any time of day or night receive prompt, safe, and effective urgent care appropriate to their needs and preferences. | 4.490  (0.7842) | **90.2*** | 4.216  (0.8322) | **82.3*** | 4.081  (0.8621) | **46.3*** |
| NHF 5.3 | The team uses clinical care protocols. | 4.471  (0.6739) | **90.2*** | 4.137  (0.8251) | **76.5*** | 3.892  (0.9063) | 70.2 |
| NHF 5.8 | Information about the patient is shared by all professionals involved in the care process. | 4.471  (0.7308) | **90.2*** | 4.157  (0.784) | **76.5*** | 4.108  (0.8091) | **56.3*** |
| NHF 9.2 | There is a procedure and utilization rules in place for adding information to the clinical record. | 4.549  (0.6727) | **90.2*** | 4.353  (0.8677) | **84.3*** | 4.216  (0.8211) | 81 |
| NICE 2.1 | People approaching the end of life receive communication and information in an accessible and sensitive way in response to their needs and preferences. | 4.451  (0.7567) | **88.2*** | 4.098  (0.9435) | **74.5*** | 3.892  (0.9364) | **68.5*** |
| NICE 2.2 | Families and carers of people approaching the end of life receive communication and information in an accessible and sensitive way in response to their needs and preferences. | 4.510  (0.7035) | 88.2 | 4.216  (0.8789) | 48.4 | 4.081  (0.8938) | 46.3 |
| NHF 12.2 | The professionals on the team safeguard the rights, responsibilities, and safety of the patient. | 4.608  (0.6951) | **88.2*** | 4.333  (0.8165) | **82.3*** | 4.189  (0.8445) | 78.3 |
| NHF12.2.1 | The team informs both the patient and his or her legal guardian of the patient’s rights. | 4.412  (0.8758) | **88.2*** | 4.020  (1.1044) | **72.5*** | 3.811  (1.17471) | 70.2 |
| NHF 5.4 | The team has a statement of the rights and guarantees of patients and families available. | 4.294  (0.8785) | 86.3 | 3.902  (1.0818) | 68.6 | 3.378  (1.2099) | 54 |
| NICE 3.2 | A personalized care plan for people approaching the end of life which is appropriate to their needs and preferences is developed and reviewed. | 4.471  (0.8088) | **71.2*** | 4.02  (1.048) | **76.5*** | 3.730  (1.1462) | **67.5*** |
| NHF 12.3.1 | The professionals on the team ask the patient and family members how they would like to be informed about the diagnosis/prognosis/treatment progress of the disease and reflect this in the clinical record in a clearly visible place. | 4.353  (0.955) | **84.3*** | 3.922  (1.0362) | **70.6*** | 3.622  (1.1631) | **59.4*** |
| NHF 12.3 | The team enables the patient to be involved in decision-making throughout the course of the disease. | 4.529  (0.7577) | 88.3* | 3.961  (1.0190) | 70.2* | 3.784  (1.0836) | 64.8* |
| NHF 12.3.2 | There is an education program for patients and families who wish to use it, which facilitates decision-making throughout the course of the disease. | 4.078  (1.0741) | 74.5 | 3.392  (1.1845) | 45.1 | 2.541  (1.0953) | 21.6 |
| NHF 12.4.10 | The team provides information on the benefits and adverse effects of the treatments that may be provided to the patient. | 4.471  (0.7837) | **90.2*** | 4.314  (0.8364) | **84.3*** | 3.89  (0.8819) | **69.2*** |
| NHF 12.7 | There is an advance care directive document in place. | 4.353  (1.0738) | 82.4* | 4.098  (1.1875) | **74.5*** | 3.135  (1.4750) | 43.2* |
| NHF 5.2 | Patient referral criteria are clearly defined. | 4.510  (0.7582) | **88.2*** | 4.137  (0.9596) | **72.6*** | 3.757  (1.0112) | **64.8*** |
| NHF 11 | People approaching the end of life are identified in a timely manner and receive coordinated care according to a personalized care plan, including prompt access to comprehensive support, equipment, and medication management. | 4.510  (0.7035) | **88.2*** | 4.118  (0.8636) | **72.6*** | 3.946  (0.9112) | 72.9 |
| NHF 12.5.8 | Protocols and clinical guidelines for providing education and information about the dying phase to the family are available to the team. | 4.314  (0.972) | 82.4 | 3.706  (1.2049) | 60.8 | 3.135  (1.13) | 40.5 |
| NICE 12 | The body of a person who has died is cared for in a culturally sensitive and dignified manner. | 4.725  (0.6026) | **96*** | 4.725  (0.4931) | **98*** | 4.622  (0.5940) | 94.6 |
| NICE 13 | Families and carers of people who have died receive timely verification and certification of the death. | 4.784  (0.4610) | **98*** | 4.706  (0.5402) | **96.1*** | 4.676  (0.5299) | 97.3 |
| NICE 14 | People closely affected by a death are communicated with in a sensitive way. | 4.784  (0.4610) | **98*** | 4.608  (0.6249) | **92.1*** | 4.486  (0.6065) | 94.6 |
| NHF 12.5 | Protocols and clinical guidelines for providing grief care are available to the team. | 4.353  (0.9127) | 84.4 | 3.941  (1.0661) | 68.7 | 3.081  (1.1396) | **37.8** |
| NICE 14 | Families of the deceased are offered emotional and spiritual support appropriate to their needs and preferences during the grieving process. | 4.412  (0.8758) | **86.3*** | 3.902  (0.9645) | **70.6*** | 3.459  (1.1204) | **51.3*** |
