# Supplementary material for: NUrsing Homes End of Life care Program (NUHELP): developing a complex intervention
Source: BMC Palliat Care. 2021 Jun 26;20:98. doi: 10.1186/s12904-021-00788-1 (PMC8234765; doi:10.1186/s12904-021-00788-1)
Supplement: Supplementary file 2 — Additional file 2. Example of NUHELP Intervention. [file 12904_2021_788_MOESM2_ESM.docx]

INTERVENTION

“EXPLORING PATIENTS’ PREFERENCES REGARDING THE INFORMATION THEY WISH TO RECEIVE”

Definition: To identify users’ preferences about the type and amount of information they wish to receive.

INTERVENTION COMPONENTS:

1.-A semi-structured interview is carried out to ascertain patients’ preferences regarding the information they wish to receive about their clinical status.

APPENDIX: Example of an interview aiming to identify information needs AMONG PATIENTS (1) ⁠

• Are you aware of your current clinical status?

• How much information regarding your clinical status do you wish to receive: no additional information/some additional information/as much information as possible?

• If you wish to continue receiving information, which of the following topics would you like more information about?

◦ Medical diagnosis or diagnoses.

◦ Your current clinical stage.

◦ Organ involvement.

◦ Current and potential future symptoms.

◦ Steps required to manage symptoms.

◦ Therapeutic options for your condition(s) and their possible side effects.

◦ Prognosis for your condition(s), the possibility of a cure, etc.

◦ Ways to cope psychologically with your condition(s).

• If you wish to continue receiving information, how much information would you like to receive?

◦ All available information.

◦ Only the most positive or pleasant information.

◦ Only the information necessary to ensure you receive proper care.

◦ As much information as possible.
